# The rediscovery and redescription of the holotype of the Late Jurassic turtle *Plesiochelys etalloni*

**DOI:** 10.7717/peerj.258

**Published:** 2014-02-06

**Authors:** Jérémy Anquetin, Sylvie Deschamps, Julien Claude

**Affiliations:** 1 Section d’archéologie et paléontologie, Office de la culture, République et Canton du Jura, Porrentruy, Switzerland; 2 UMR CNRS 7207 MNHN UPMC, Muséum national d’Histoire naturelle, Paris, France; 3 Musée d’Archéologie du Jura, CCE René Rémond, Lons-le-Saunier, France; 4 Institut des Sciences de l’Evolution de Montpellier, UMR 5554 CNRS, Montpellier, France

**Keywords:** Plesiochelys, Plesiochelyidae, Testudines, Kimmeridgian, Tithonian, Late Jurassic, Switzerland, France

## Abstract

Plesiochelyidae are a major component of Late Jurassic shallow marine environments throughout Europe. However, the taxonomy of plesiochelyid turtles is rather confused. Over the years, many taxa have been synonymized with *Plesiochelys etalloni*, one of the first described species. However, the holotype of *P. etalloni* (and only specimen known from Lect, the type locality) was lost for more than 150 years. This specimen has been recently rediscovered in the collections of the Musée d’archéologie du Jura in Lons-le-Saunier, France. For the first time since its original description in 1857, the holotype of *P. etalloni* is redescribed and compared to relevant material. The taxonomic status of this taxon is revised accordingly. Based on the morphology of the newly rediscovered holotype and on a reassessment of specimens from Solothurn (Switzerland), the species *P. solodurensis*, *P. sanctaeverenae* and *P. langii* are synonymized with *P. etalloni*. Known skull-shell associations for *P. etalloni* are re-evaluated in light of the new morphological information available since the rediscovery of this holotype specimen. Finally, we confirm that *Plesiochelys* is represented by a single species in the Late Jurassic of the Jura Mountains.

## Introduction

Despite numerous historical discoveries dating from as early as the beginning of the nineteenth century (e.g., Cuvier, 1824; Pictet & Humbert, 1857; von Meyer, 1860; Pictet, 1860; Wagner, 1861; Maack, 1869; Rütimeyer, 1873), the diversity of Late Jurassic European turtles still eludes our understanding. Traditionally referred to the families Plesiochelyidae Baur, 1888, Thalassemydidae Zittel, 1889, and Eurysternidae Dollo, 1886, these forms are generally considered to be basal eucryptodires, but their exact relationships with one another and with other turtle groups remain largely unclear and usually vary among authors (e.g., Gaffney & Meylan, 1988; Hirayama, Brinkman & Danilov, 2000; Gaffney *et al.*, 2007; Joyce, 2007; Sterli, 2010; Rabi *et al.*, 2013). A number of reasons may be invoked to explain this situation, but at least two of these are the much needed revision of the rich historical material and the limited number of skull-shell associations. Cranial characters are important for turtle systematics, yet many Late Jurassic turtles from Europe are known from postcranial or cranial material only. There are few exceptions however, for which both the skull and the shell are well known: notably *Solnhofia parsonsi* Gaffney (1975*b*) and *Plesiochelys etalloni* (Pictet & Humbert, 1857).

*Emys etalloni* Pictet & Humbert (1857) was described based on a single shell found in the French Jura Mountains (see below). A few years later, Rütimeyer (1873) reassigned this species to his newly created genus *Plesiochelys*. The type species of *Plesiochelys* is *P. solodurensis* Rütimeyer (1873), a species typified based on material from the prolific quarries near Solothurn in the Swiss Jura Mountains. Rütimeyer (1873) and Bräm (1965) afterwards both recognized the presence of *P. solodurensis* and *P. etalloni* in the Solothurn deposits. Although turtle skulls were known in Solothurn since as early as the 1820s (Cuvier, 1824; see Bräm, 1965 for an historical account), they were not fully prepared until the 1970s (Gaffney, 1975*a*). Based on this material, Gaffney (1975*a*) concluded that *Emys etalloni* Pictet & Humbert (1857), *Emys jaccardi* Pictet (1860) from the Late Jurassic of Les Hauts-Geneveys (Canton of Neuchâtel, Switzerland), *Stylemys lindenensis* Maack (1869) from the Kimmeridgian of Hanover (Germany), as well as *Plesiochelys solodurensis* Rütimeyer (1873), *Plesiochelys sanctaeverenae* Rütimeyer (1873), *Craspedochelys picteti* Rütimeyer (1873), and *Craspedochelys crassa* Rütimeyer (1873) from the late Kimmeridgian of Solothurn (Canton of Solothurn, Switzerland) represented a single species, which should be named *Plesiochelys etalloni* (Pictet & Humbert, 1857) in application of the Principle of Priority. The immediate effect was that *P. etalloni* was henceforth included into phylogenetic analyses, which helped to improve our understanding of the systematics and relationships of Late Jurassic and Early Cretaceous turtles from Europe and Asia (e.g., Gaffney & Meylan, 1988; Hirayama, Brinkman & Danilov, 2000; Joyce, 2007). However, this relatively inclusive synonymy list was not generally accepted among specialists (including ourselves). For example, several subsequent authors still considered *C. picteti*, *C. jaccardi*, *P. etalloni*, and *P. solodurensis* as different species (e.g., Antunes, Becquart & de Broin, 1988; Lapparent de Broin, Lange-Badré & Dutrieux, 1996).

This extremely confusing situation is in part due to the fact that the holotype of *Plesiochelys etalloni* was considered to be lost since the 1860s and was therefore unavailable notably to Rütimeyer (1873), Bräm (1965), Gaffney (1975*a*), and Lapparent de Broin, Lange-Badré & Dutrieux (1996). These authors based their conclusions on the original description (Pictet & Humbert, 1857) and on plaster casts of the type specimen. These casts are available in several European museums, notably in Paris, Geneva, Lons-le-Saunier, Montbéliard, and Besançon, but they differ among each other regarding quality and completeness. Some of these casts lack the pygal region of the carapace (e.g., the Geneva copy), whereas others only consist of a cast of the carapace (e.g., the Montbéliard copy). They do not allow a precise examination of sutures and sulci, especially on the carapace where some sutures must be determined from their imprints on the sediment. In contrast, we have been fortunate to locate this historical specimen in the collections of the Musée d’archéologie du Jura in Lons-le-Saunier, France. We have also been able to retrace the history of this specimen as it passed from one owner to the other. This material is redescribed herein and the taxonomic status of *Plesiochelys etalloni* is revised accordingly. This specimen also gives us the opportunity to reassess the validity of several species from the Late Jurassic of the Jura Mountains. Finally, this rediscovery allow us to re-evaluate the known skull-shell associations for *P. etalloni*.

**Institutional abbreviations:** **MAJ**, Musée d’archéologie du Jura, Lons-le-Saunier, France; **MH**, Naturhistorisches Museum, Basel, Switzerland; **NMS**, Naturmuseum Solothurn, Switzerland.

## Historical Background

Pictet & Humbert (1857) explained that the holotype of *Plesiochelys etalloni* was collected by a local priest in the forest close to the village of Lect, near Moirans-en-Montagne (Jura, France). When they studied the specimen, it was in possession of Joseph Célestin Girod, vicar general of the Saint-Claude diocese (France). Neither Rütimeyer (1873) nor Bräm (1965) gave indication relative to the repository of this specimen. Gaffney (1975*a*) indicated that H. Bräm told him the specimen was lost. Lapparent de Broin, Lange-Badré & Dutrieux (1996) explained that they actively looked for the type without success, but they figured the plaster cast housed in the Natural History Museum in Geneva, Switzerland. Finally, without further explanation, Lapparent de Broin (2001) stated that the holotype of *P. etalloni* had been located in the Natural History Museum of Besançon, France. After verification, it appears that this information is incorrect.

**Figure 1:**
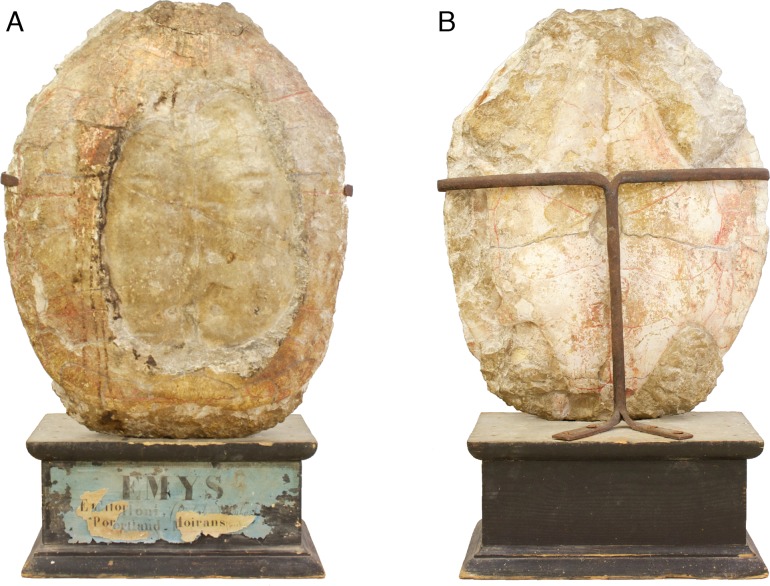
Specimen mounted on a wooden pedestal. MAJ 2005-11-1, holotype of *Plesiochelys etalloni* (Pictet & Humbert, 1857). Specimen mounted on a wooden pedestal with the old label “*Emys etalloni*, (Pictet et Humbert) - Portland - Moirans (Jura)”. (A) carapace; (B) plastron. Note that the specimen is upside down.

**Figure 2:**
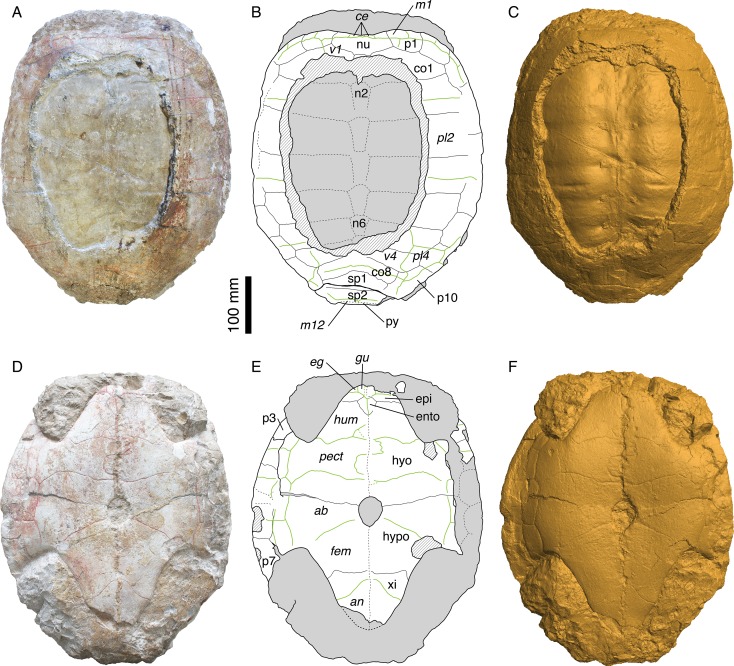
Morphology of the holotype. MAJ 2005-11-1, holotype of *Plesiochelys etalloni* (Pictet & Humbert, 1857). (A) photograph of the carapace; (B) interpretative drawing of the carapace; (C) 3D surface reconstruction of the carapace; (D) photograph of the plastron; (E) interpretative drawing of the plastron; (F) 3D surface reconstruction of the plastron. Bones are white; stripped lines indicate internal bone layers; green solid lines indicate scale sulci; matrix is gray. Abbreviations: *ab*, abdominal; *an*, anal; *ce*, cervical; co, costal; *eg*, extragular; epi, epiplastron; ento, entoplastron; *fem*, femoral; *gu*, gular; hyo, hyoplastron; hypo, hypoplastron; *hum*, humeral; *m*, marginal; n, neural; nu, nuchal; p, peripheral; *pect*, pectoral; *pl*; pleural; py, pygal; sp, suprapygal; *v*, vertebral; xi, xiphiplastron.

One of us (SD) rediscovered the original specimen a few years ago in the collections of the Musée d’archéologie du Jura in Lons-le-Saunier, France. Examination leaves no doubt whatsoever on the identity of this specimen (Figs. 1 and 2). This specimen (MAJ 2005-11-1) was not always housed at the MAJ: it was donated to the museum by a private owner in 1994. The MAJ also houses a plaster copy of the fossil, which was offered by C-A Etallon, the renowned geologist, on March 30th, 1857. After a careful investigation, we were able to uncover most of the history of the fossil shell before it was finally donated to the MAJ.

The claim that the holotype of *P. etalloni* was housed in the Natural History Museum of Besançon (Lapparent de Broin, 2001) is not entirely incorrect. We have found evidence that the specimen was actually part of the Besançon Museum collection, if only for a short time. This part of the story can be followed in the *Mémoires de la Société d’émulation du département du Doubs* dated from 1859 and 1860. Bishop Mabile, Vicar Girod’s superior, offered the specimen to Mr Thiébaud, a member of the Société d’émulation du Doubs (a French scientific society), who gave it to the Besançon Museum. The exact date is unclear, but it was sometime between 1857 and 1859. In 1859, Vicar Girod wrote to the French Minister of Public Education and Cults, who turned him down, then to the Rector explaining that he had never agreed for the fossil to be given for free to the Besançon Museum and claimed property on the specimen. The Rector abided and the holotype of *P. etalloni* was sent back to Saint-Claude (Jura, France). Joseph Célestin Girod died in 1863 and the track of the specimen was lost.

The last piece of the puzzle was revealed when Mr and Mrs Lacroix donated the specimen to the MAJ in 1994. After claiming the fossil as his own, the Vicar sold it to a private party, the ancestor of Mr and Mrs Lacroix, in order to finance the renovation of his church. The transaction must have occurred between 1859 and 1863. Until 1994, the holotype of *P. etalloni* remained in this family and was passed from one generation to another (Fig. 1).

## Systematic Paleontology

 TESTUDINES Batsch, 1788

 EUCRYPTODIRA Gaffney, 1975*c*

 PLESIOCHELYIDAE Baur, 1888

Remarks.—Late Jurassic eucryptodires from Europe are traditionally referred to one of the three following families: Plesiochelyidae, Eurysternidae, and Thalassemydidae. These names have been inconsistently, but regularly, used since the nineteenth century. Bräm (1965) conceptualized these families as follows. Plesiochelyids were considered to be characterized by a completely ossified carapace, an osseous bridge, and the absence of lateral plastral fontanelles. Eurysternids were characterized by an incompletely ossified carapace (presence of costo-peripheral fontanelles), a ligamentous bridge, and the presence of both lateral and central plastral fontanelles. Finally, thalassemydids were considered to retain costo-peripheral fontanelles and to lack lateral plastral fontanelles. In addition to these characters, the presence of three cervical scales has been regularly mentioned as a distinctive feature of plesiochelyids (e.g., Lapparent de Broin, Lange-Badré & Dutrieux, 1996; Danilov, 2005; Pérez-García, *in* *press*), although some eurysternids are known to share this characteristic (Bräm, 1965; Joyce, 2003; Anquetin & Joyce, *in* *press*). This and other accumulating evidence (J Anquetin, unpublished data) suggest that the three aforementioned families may actually form a monophyletic group. As such, the definition of Plesiochelyidae is likely to change in the near future. That being said, we will continue to use the traditional definition of Plesiochelyidae (sensu Gaffney, 1975*a*; Lapparent de Broin, Lange-Badré & Dutrieux, 1996; Danilov, 2005) for the purpose of the present study. Consequently, *Plesiochelys etalloni* is considered a member of the Plesiochelyidae based on the presence of three cervical scales and the absence of carapacial and lateral plastral fontanelles.

*Plesiochelys* Rütimeyer, 1873

*Plesiochelys etalloni* Pictet & Humbert, 1857

*Emys Etalloni*Pictet & Humbert, 1857 (original description)

*Plesiochelys solodurensis*Rütimeyer, 1873 (subjective synonymy)

*Plesiochelys sanctaeverenae*Rütimeyer, 1873 (subjective synonymy)

*Plesiochelys langii*Rütimeyer, 1873 (subjective synonymy)

Type material.—MAJ 2005-11-1, a shell missing a large part of the carapace medially. Holotype by monotypy.

Type horizon and locality.—“Forêt de Lect” (Lect is a small village) near Moirans-en-Montagne (Department of Jura, France), Late Jurassic. The exact horizon is uncertain, but most outcrops in the vicinity of Lect are either Kimmeridgian or early Tithonian in age. According to Etallon (1857), the specimen was found in the “calcaires portlandiens”. *Gravesia gigas* was also found in these limestones (Etallon, 1857), which led Lapparent de Broin, Lange-Badré & Dutrieux (1996) to conclude that MAJ 2005-11-1 was probably from the early Tithonian.

Illustrations of type.—(Pictet & Humbert, 1857: plates I-III); Figs. 1 and 2.

Referred specimens.—See Bräm (1965): specimens referred to *P. etalloni*, *P. solodurensis*, *P. sanctaeverenae*. For cranial material, see Gaffney (1975*a*).

Revised diagnosis.—Species of Plesiochelyidae (sensu Gaffney, 1975*a*; Lapparent de Broin, Lange-Badré & Dutrieux, 1996; Danilov, 2005) known from most of the skeleton. Differing from *P. planiceps* (Owen, 1842) in the following features (see Gaffney, 1975*a*): lingual ridge of maxilla usually lower than in *P. planiceps*; anterior portion of lingual ridge on the lower jaw curving anteriorly (as opposed to medially in *P. planiceps*); at level of vomer-premaxilla suture the distance between lingual ridges of maxillae is narrower than in *P. planiceps*. Differing from all other *Plesiochelys* species in the following combination of features: relatively large carapace (up to 550 mm in length); shell bones relatively thick; carapace oval in outline; wide and shallow nuchal notch; additional trapezoidal element often present between the neural series and first suprapygal; wide vertebral scales, usually extending approximately half the length of the costals; anterior marginal scales very short and not extending onto costals; relatively long plastron (85–90% of carapace length) sutured to the carapace along a long osseous bridge; entoplastron variable in size, usually diamond-shaped with a more or less extended posterior part; hyoplastron and xiphiplastron longer than wide; central plastral fontanelle retained in some adults; short gular and extragular scales; gular-humeral sulcus reaching the anterior part of the entoplastron; long humeral scale; four inframarginal scales mostly covering the plastral elements.

Remarks.—The synonymy list is intentionally restricted to the *Plesiochelys* species described by Rütimeyer (1873) and later revised by Bräm (1965). The synonymy list proposed by Gaffney (1975*a*) is more inclusive, but testing it would require an extensive revision of historical material at the European scale, something that was done neither by Gaffney (1975*a*), 1976 nor any subsequent author (see Discussion) and that is beyond the scope of the present study as well.

## Description

### General description

The holotype of *Plesiochelys etalloni* (MAJ 2005-11-1) is a large, oval shell with carapace and plastron still articulated (Fig. 2 and Video S1). The specimen may have been slightly flattened during fossilization, but there are no indications of severe deformation. The specimen is fairly complete, although part of the left bridge and central part of the carapace are missing. The part of the carapace that is missing reveals the steinkern, which probably explains why the locals regarded this specimen as the imprint of a human torso (Pictet & Humbert, 1857). There are some indications in the right axillary and inguinal notches that some elements of the appendicular skeleton are preserved within the matrix, but as it stands these elements are undetermined.

### Carapace

As preserved, the length of the carapace is 471 mm, but most of the pygal is missing (Figs. 2A–2C and S2). The carapace is evenly oval in outline, except anteriorly where there is a broad, shallow nuchal notch. A large part of the carapace is missing centrally. As a result, the neural series and the medial half of most costals are only visible as imprints on the steinkern.

The nuchal is a wide and trapezoidal element. The nuchal notch is shallow, but it extends laterally on the medial part of the first peripheral. Only the anterior part of the first neural is preserved. This element was apparently longer than wide and rectangular. Neurals 2–6 are preserved as imprints on the steinkern. They are elongate, hexagonal elements with their shorter sides facing anteriorly. The sixth neural is shorter than the previous elements in the series. Behind the sixth neural, the imprint of the anterior part of the seventh neural is also preserved. Posteriorly, the steinkern is covered by the bony carapace, but the sutures is this area are hardly visible and it is uncertain whether or not there are additional elements to the neural series. Although it is impossible to be certain, the eighth costals may contact one another in the midline. Most specimens from Solothurn referred to *P. etalloni* (sensu this study) have an eighth neural and an additional trapezoidal element of uncertain identity (additional neural, additional suprapygal, or neomorphic bone) between the seventh neural and the first suprapygal. This area is however relatively variable in plesiochelyids and other basal eucryptodires, and neurals 7 and or 8 may be reduced or lost allowing a medial contact of costals 7 and/or 8 (Bräm, 1965; Pérez-García, 2012; J Anquetin, unpublished data). There are eight pairs of costals. The first costal is relatively short compared to the following ones. Anteriorly, it contacts the nuchal and the three first peripherals. Costals 2–4 are wider and longer elements, with costal 3 being notably wide distally. Costals 5–8 decrease progressively in length and width. There were certainly 11 pairs of peripherals, even if they cannot be clearly all observed on the fossil. The sutures between peripherals 4, 5 and 6 are not preserved dorsally, but they are visible ventrally. Posteromedially, the suture between the tenth and eleventh peripherals is not preserved. Peripherals are longer than wide, rectangular elements. Most of peripheral 11 is missing on both sides. The posteromedial region of the carapace is rather poorly preserved. There are two large suprapygals. The first suprapygal is a broad element that contacts the eighth costals anteriorly along a long, anteriorly concave suture and the second suprapygal posteriorly along a more or less straight suture (poorly preserved). Laterally, the first suprapygal appears to contact only the eleventh peripheral. The exact outline of the second suprapygal is uncertain, because most of its sutures with surrounding elements are effaced. Posteriorly, just in front of the broken margin of the carapace, the suture with the pygal is barely discernible.

Three cervical scales are clearly visible on the nuchal. Plesiochelyids have long been thought to be characterized by this character, but its distribution is actually wider. For example, several eurysternids are known to have three cervical scales (Bräm, 1965; Joyce, 2003; Anquetin & Joyce, *in* *press*). Scale sulci are clearly apparent on the carapace, but very little can be said about the vertebral scales because a large part of the carapace is missing. The first vertebral scale is a broad element, wider anteriorly than posteriorly. Its lateral margins extends on the first costal and first peripheral, but not on the nuchal. Laterally, the first vertebral scale reaches the lateral part of the first marginal. Nothing can be said about the second and third vertebral scales. The fourth vertebral scale is a broad element extending laterally about two-thirds of the length of the sixth and seventh costals. The outline of the fourth vertebral scale is somewhat unusual. Posterolaterally, its lateral margin extends abruptly onto the tenth peripheral. This unusual shape is symmetrical, but, based on our experience of the intraspecific variability in plesiochelyids, we grant it no systematic value. The fifth vertebral scale is a wide, pentagonal element extending onto costals 8, suprapygals 1 and 2, and peripherals 10 and 11. There are four pleural scales. The outlines of pleurals 1–3 are uncertain. The first pleural scale contacts marginals 1–4 and maybe also the fifth marginal scale. The first pleural scale is slightly shorter than pleurals 2 and 3. The second pleural scale reaches the seventh marginal scale posteriorly on the sixth peripheral. The fourth pleural scale is a reduced element covering only a small portion of the sixth and seventh costals and the medial part of the ninth and tenth peripherals. Marginals are only partly preserved. Marginals 1–6 are still partly visible on the right anterolateral part of the carapace. When preserved, the pleuro-marginal sulci are always on the peripherals and never extend onto the costals. It should also be noted that the last marginal scales (probably the twelfth pair, although it is impossible to be sure) extend anteriorly onto the second suprapygal.

### Plastron

The plastron of MAJ 2005-11-1 is mostly complete (Figs. 2D–2F and S3). The anterior margin of the left epiplastron, the bridge area on the left hand side, and posterior tip of the xiphiplastra are broken. The matrix preserved the imprints of the broken parts of the bridge and xiphiplastra. The total length of the plastron is 431 mm, measuring from the epiplastra anteriorly to the imprint of the xiphiplastra posteriorly. As such, the plastron represents 91.5% of the length of the preserved carapace (the true ratio would be slightly lower if the pygal had been preserved). The plastron is strongly sutured to the carapace. The bridge extends from the posterior part of the third peripheral to the anterior part of the eighth. The axillary and inguinal notches are deep. A small central fontanelle is present between the hyo- and hypoplastra. The anterior lobe is shorter than the posterior lobe, which is itself shorter than the bridge measured between the axillary and inguinal notches. The anterior lobe is trapezoidal in outline with a nearly straight anterior margin. The posterior lobe has a triangular outline with a slightly rounded posterior tip (no anal notch). The central part of the plastron is slightly concave. This concavity may have been natural.

None of the two epiplastra is complete. The left one is missing its anterior margin, whereas the lateral part of the right one is partly covered by matrix. As preserved, the epiplastra are relatively short, wider than long elements. They contact one another medially, the hyoplastra posteriorly, and the entoplastron posteromedially. The epi-hyoplastron suture is straight and transverse. The entoplastron is a diamond-shaped, slightly longer than wide element with its posterior faces slightly more elongated than the anterior. The hyoplastron is a large, longer than wide element. Posteromedially, the hyoplastron forms the anterior third of the central plastral fontanelle. The hyo-hypoplastral suture is slightly concave anteriorly, more so medially. The hypoplastron is shorter than the hyoplastron. It forms the remaining two-thirds of the central fontanelle. The suture between the hypoplastron and the xiphiplastron is mostly straight and transverse medially. Laterally, its bends suddenly backwards, as it is so often seen in turtles. The xiphiplastron is a triangular, longer than wide element with a slight broadening where the femoro-anal sulcus meets its lateral margin, as correctly noted by Pictet & Humbert (1857). The midline contacts between the different plastral elements are partly disarticulated (Fig. S3), so that the exact position of the sutures is difficult to assess. Probably as a result, Pictet & Humbert (1857) erroneously described and depicted a very small fontanelle between the hypo- and xiphiplastra. Direct examination of the specimen and observation of the 3D surface reconstruction (Video S1 and Fig. S3) both suggest that there is no such fontanelle in MAJ 2005-11-1.

Gular and extragular scales are relatively small. The gular scales extend only a little onto the anteromedial part of the entoplastron. The extragular scales are restricted to the epiplastra. The long humeral scales cover the rest of the anterior plastral lobe. The pectoral scale is nearly as long as the abdominal scale on the midline, but both are shorter than the humeral scale. The abdominal-femoral sulcus is oblique and extends from the inguinal notch to the posterior third of the central plastral fontanelle. The femoral is the longest scale of the plastron. The femoral-anal sulcus is deeply concave posteriorly in its medial part. The anal scales are restricted to the xiphiplastra. The medial sulcus between paired scales is unusually irregular. The median sulcus diverges strongly from the midline between the humeral and pectoral scales, being notably sinusoidal between the latter. The median sulcus is more poorly preserved between the femoral and anal scales, but observation of the 3D surface reconstruction (Video S1) suggests that it might also have been slightly sinusoidal, at least in the posterior part of the femoral scales. The bridge area is covered by four inframarginal scales increasing in length posteriorly. The two first and the last are restricted to the hyoplastron and hypoplastron, respectively. The third inframarginal scale covers the hyoplastron anteriorly, the hypoplastron posteriorly, and a small portion of the fifth peripheral laterally.

## Discussion

### Skull-shell associations

Despite a profusion of material collected from the Late Jurassic of Europe, relatively few species are known from both skull and shell material. European lithographic limestone localities (especially Solnhofen, Kelheim, and Cerin) have produced a fair number of relatively complete, articulated specimens with shell, skull, and various elements of the skeleton (e.g., von Meyer, 1860), but the cranial material is always badly crushed and very difficult to interpret. Hence, the skull is ‘known’ in some eurysternids, such as *Eurysternum wagleri*, *Idiochelys fitzingeri*, and *Palaeomedusa testa* (e.g., Jourdan, 1862; Joyce, 2003; Anquetin & Joyce, *in* *press*), but only scarce information can be gathered from these examples.

Among European Late Jurassic turtles, only *Solnhofia parsonsi* and *Plesiochelys etalloni* are sufficiently known from both skull and shell material. *Solnhofia parsonsi* was described by Gaffney (1975*b*) based on two isolated skulls, one from the Solnhofen region (Germany), one from Solothurn (Switzerland). Later, Joyce (2000) described a nearly complete skeleton that can be confidently referred to *S. parsonsi*. Additional skull and associated fragmentary shell remains were described by Rieppel (1980) and assigned to *Thalassemys moseri* Bräm (1965), but the validity of both this taxon and this referral was questioned by subsequent authors (e.g., Gaffney & Meylan, 1988; Lapparent de Broin, Lange-Badré & Dutrieux, 1996). This material should therefore be revised.

**Figure 3:**
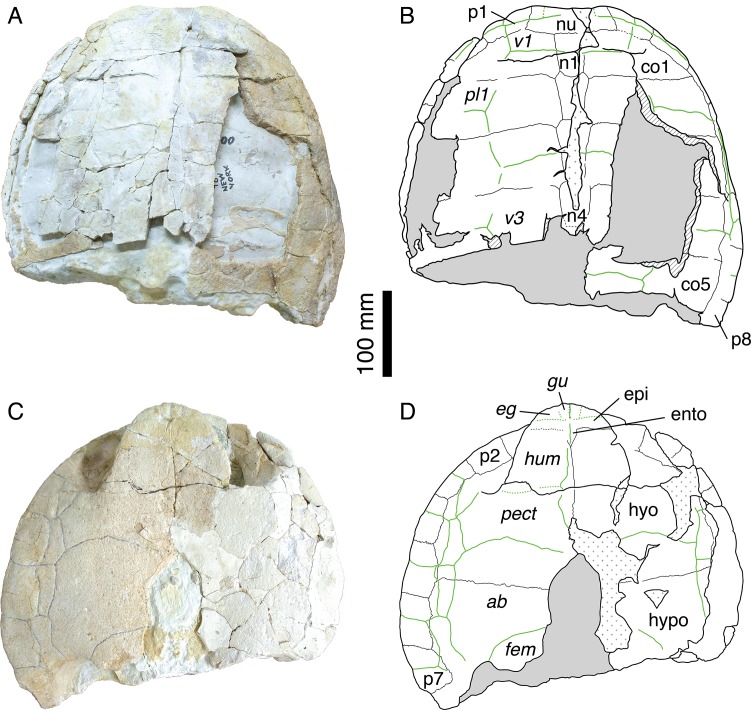
Shell of specimen MH 435. MH 435, *Plesiochelys etalloni* (Pictet & Humbert, 1857). (A) photograph of the carapace; (B) interpretative drawing of the carapace; (C) photograph of the plastron; (D) interpretative drawing of the plastron. Bones are white; stripped lines indicate internal bone layers; green solid lines indicate scale sulci; dotted areas indicate reconstructed parts; matrix is gray. Abbreviations: *ab*, abdominal; co, costal; *eg*, extragular; epi, epiplastron; ento, entoplastron; *fem*, femoral; *gu*, gular; hyo, hyoplastron; hypo, hypoplastron; *hum*, humeral; n, neural; nu, nuchal; p, peripheral; *pect*, pectoral; *pl*; pleural; *v*, vertebral.

Skulls of *P. etalloni* are known since the early nineteenth century (e.g., Cuvier, 1824; Rütimeyer, 1873; Bräm, 1965), although they were not necessarily assigned to this species in those times. The Solothurn Turtle Limestone has produced four *Plesiochelys* skulls, which Gaffney (1975*a*) identified as belonging to a single species. Among these four skulls, only one (NMS 594) is associated with significant shell material (few disarticulated costals and peripherals and partial posterior half of a plastron). Bräm (1965) identified this specimen as *P. etalloni* based on the probable presence of a central plastral fontanelle. However, this material is too fragmentary to allow a definitive specific identification. Only one other skull-shell association exists for *P. etalloni*. It is a specimen (MH 435) that was found in the Kimmeridgian near Glovelier (Canton of Jura, Switzerland). Bräm (1965) referred this material to *P. etalloni* without further description and depicted the skull and a humerus (ibid.: plate 4, Figs. 1–4). The skull, one of the best for *P. etalloni*, was subsequently prepared and Gaffney (1975*a*) followed the identification of Bräm (1965). Gaffney (1975*a*;7) examined the associated, incompletely prepared shell material and concluded that “the shell features as determinable at this time are consistent with [his] concept of *Plesiochelys etalloni*”. Because Gaffney’s (1975*a*) concept of *P. etalloni* is inclusive and not necessarily accepted among fossil turtle specialists, it was important to reassess the shell material of MH 435 and compare it with the newly rediscovered holotype specimen of *P. etalloni*.

If the skull of MH 435 has been extensively studied (Gaffney, 1975*a*, 1976; Sterli, 2010; Carabajal *et al.*, 2013), the associated shell material has never been described or illustrated. This material (Fig. 3) consists of the anterior half of a shell with carapace and plastron still in articulation. Everything posterior to the fifth costal on the carapace and inguinal notch on the plastron is missing. Both the carapace and the plastron are greatly fractured. Many fragments of the costals are missing. The carapace is oval in outline with a broad, shallow nuchal notch (Figs. 3A–3B). The nuchal is a wide and trapezoidal element. The first neural is rectangular, whereas neurals 2–4 are hexagonal with their shorter sides anteriorly. Cervical scale sulci are not preserved. The first vertebral scale is a broad, trapezoidal element that extends laterally onto the first peripheral and contacts the lateral border of the first marginal scale. The second and third vertebral scales are wide and hexagonal. Their sulci are moderately sinuous, as it is common in Solothurn specimens referred to *P. etalloni* (Bräm, 1965). The anterior margin of the anterior lobe of the plastron is rounded (Figs. 3C–3D). The epiplastron is separated from the hyoplastron by a straight, transverse suture. The hyoplastron is longer than wide. There is an oval central fontanelle between the hyo- and hypoplastron. The hyo-hyoplastral suture is relatively straight and slightly oblique defining a small concavity toward the anterior. The bridge is long and osseous. It extends from the posterior half of the third peripheral to the anterior part of the eighth peripheral. The scale arrangement on the plastron is similar to that of MAJ 2005-11-1. The median sulcus between the humeral and pectoral scales diverges strongly from the midline, although it is not sinusoidal as in the holotype of *P. etalloni*. There are four inframarginal scales increasing in length posteriorly. The above description indicates that the shell of MH 435 does not significantly differ from that of MAJ 2005-11-1. Therefore, MH 435 can be confidently referred to *P. etalloni* (sensu this study, not Gaffney, 1975*a*). This confirms the importance of this specimen, especially for phylogenetic reconstructions.

### Alpha taxonomy

As mentioned above, comparisons for the present study are restricted to the *Plesiochelys* species described by Rütimeyer (1873) and later revised by Bräm (1965), i.e., forms first described from the Late Jurassic of the Swiss and French Jura Mountains. Many specimens from the Late Jurassic of France, Germany, England, Spain and Portugal have afterwards been either referred to *P. etalloni* and *P. solodurensis* or assigned to new or indeterminate species, but these need to be revised thoroughly. Kuhn (1964) listed 22 species of *Plesiochelys* typified based on European material. It is far beyond the scope of the present study to revise the taxonomy of the genus *Plesiochelys*.

Another issue is the relatively inclusive synonymy list proposed by Gaffney (1975*a*), who synonymized the following species with *P. etalloni*: *Emys jaccardi*, *Stylemys lindenensis*, *P. solodurensis*, *P. sanctaeverenae*, *Craspedochelys picteti*, and *C. crassa*. *Stylemys lindenensis* is a form from the Late Jurassic of Hanover, Germany, and, along with many other specimens from the same region, it has never been properly revised since Oertel (1924). All other species but *E. jaccardi* were described based on material from Solothurn, Switzerland. *Emys jaccardi* was referred to *Plesiochelys* by Rütimeyer (1873) and Bräm (1965). In contrast, Antunes, Becquart & de Broin (1988) and Lapparent de Broin, Lange-Badré & Dutrieux (1996) referred this species to the genus *Craspedochelys* Rütimeyer (1873), which they distinguished from *Plesiochelys* by a shell as wide as long and a shortened plastron. Gaffney (1975*a*) argued that variation in shell shape, especially relative width (as used to differentiate *E. jaccardi* and *C. picteti* from *P. etalloni*), was probably the result of postmortem deformation and should not be considered for systematic purposes. The objective of the present paper is not to settle this argument. The fact is that Bräm (1965) is the last author to have thoroughly reassessed the shell morphology of these forms. Gaffney (1975*a*) focused essentially on skull description and did not describe shell morphology in detail. Lapparent de Broin, Lange-Badré & Dutrieux (1996) studied some of the Solothurn material, but they did not clearly formalized their views, instead proposing a general discussion as part of the description of new material from France. For the purpose of the present paper, we restrict our comparisons to *P. solodurensis*, *P. sanctaeverenae* and *P. langii*.

According to Rütimeyer (1873) and Bräm (1965), both *P. etalloni* and *P. solodurensis* are present in Solothurn, the type locality of *P. solodurensis*. However, Bräm (1965) himself admitted that differentiating the two species was not easy. *Plesiochelys etalloni* was supposed to produce slightly larger individuals than *P. solodurensis* and to retain a small central plastral fontanelle in the adults (Bräm, 1965). The proposed difference in size is minor (about 10%) and is not interpreted as being significant. We have scrutinized all fairly complete specimens from Solothurn referred to both *P. etalloni* and *P. solodurensis*, representing about 30 individuals. We have extensively looked for additional characters that would confirm the presence of two species (one with a central plastral fontanelle and one without), but have found none. For example, a close comparison between MAJ 2005-11-1 (holotype of *P. etalloni*) and NMS 59 (lectotype of *P. solodurensis*) reveals only little differences: the shape of the posterolateral sulcus of the fourth vertebral (probably anomalous in MAJ 2005-11-1); the very minute extension of the fourth marginal onto costal 2 in NMS 59; the central plastral fontanelle in MAJ 2005-11-1; and the extension of the anal scale onto the hypoplastron in NMS 59. Anomalous scale shape is relatively common among Solothurn turtles, especially for vertebral scales. Similarly, both the extension of the fourth marginal onto costals and the extension of the anal scale onto the hypoplastron, characters that are otherwise diagnostic for Xinjiangchelyidae (e.g., Tong *et al.*, 2012; Rabi *et al.*, 2013; Pérez-García, Gasulla & Ortega, *in* *press*), are variable in *P. etalloni* and other plesiochelyids (e.g., Pérez-García, *in* *press*). Hence, the retention of a central plastral fontanelle in adults is interpreted as an intraspecific variation of *P. etalloni*, and *P. solodurensis* is considered a subjective junior synonym of this species.

Bräm (1965) found no significant difference between NMS 123 and NMS 126, two carapaces referred to *P. langii*, and NMS 59, and therefore synonymized *P. langii* with *P. solodurensis*. We agree and similarly find no significant difference between these specimens and MAJ 2005-11-1. Consequently, *P. langii* is synonymized with *P. etalloni*. *Plesiochelys sanctaeverenae* was defined by Rütimeyer (1873) mainly based on NMS 118, a large, incomplete carapace. Bräm (1965) designated this specimen as the lectotype and considered this species as valid based on its larger size (carapace length = 565 mm) and elongate outline. However, observable characteristics do not allow to differentiate NMS 118 from others specimens we refer here to *P. etalloni*, especially neither from MAJ 2005-11-1 nor NMS 59. Concerning the outline of this specimen, Bräm (1965) was probably misled by the fact that the lateral parts of the carapace are largely missing. Consequently, *P. sanctaeverenae* is also considered a subjective synonym of *P. etalloni*.

From the above, we recognize only one species of *Plesiochelys* in the Jura Mountains: *Plesiochelys etalloni*. Although this conclusion may appear superficially similar to that of Gaffney (1975*a*), we reached it through an extensive re-evaluation of the Solothurn material and a redescription of the type material of *P. etalloni*, which was unavailable for these past 150 years. Since Gaffney (1975*a*), Gaffney (1976), we have an excellent knowledge of the cranial morphology of *P. etalloni*. Thanks to the present study, we now have a better understanding of the shell morphology and intraspecific variability of this species.

## Supplemental Information

10.7717/peerj.258/supp-1 Supplemental Information 1 3D rotation animation 3D rotation animation of specimen MAJ 2005-11-1, holotype of Plesiochelys etalloni (Pictet & Humbert, 1857). 3D surface mesh available upon request from the MAJ.

10.7717/peerj.258/supp-2 Supplemental Information 2 High resolution 3D reconstruction of the carapace High resolution 3D surface reconstruction of the carapace of MAJ 2005-11-1, holotype of Plesiochelys etalloni (Pictet &amp; Humbert, 1857). Reconstruction courtesy of and copyright David Vuillermoz, Muse d’archologie du Jura. 3D surface mesh available upon request from the MAJ.

10.7717/peerj.258/supp-3 Supplemental Information 3 High resolution 3D reconstruction of the plastron High resolution 3D surface reconstruction of the plastron of MAJ 2005-11-1, holotype of Plesiochelys etalloni (Pictet &amp; Humbert, 1857). Reconstruction courtesy of and copyright David Vuillermoz, Muse d’archologie du Jura. 3D surface mesh available upon request from the MAJ.
