# Supplementary figures and images for: The rediscovery and redescription of the holotype of the Late Jurassic turtle Plesiochelys etalloni
Source: PeerJ. 2014 Feb 6;2:e258. doi: 10.7717/peerj.258 (PMC3932733; doi:10.7717/peerj.258)

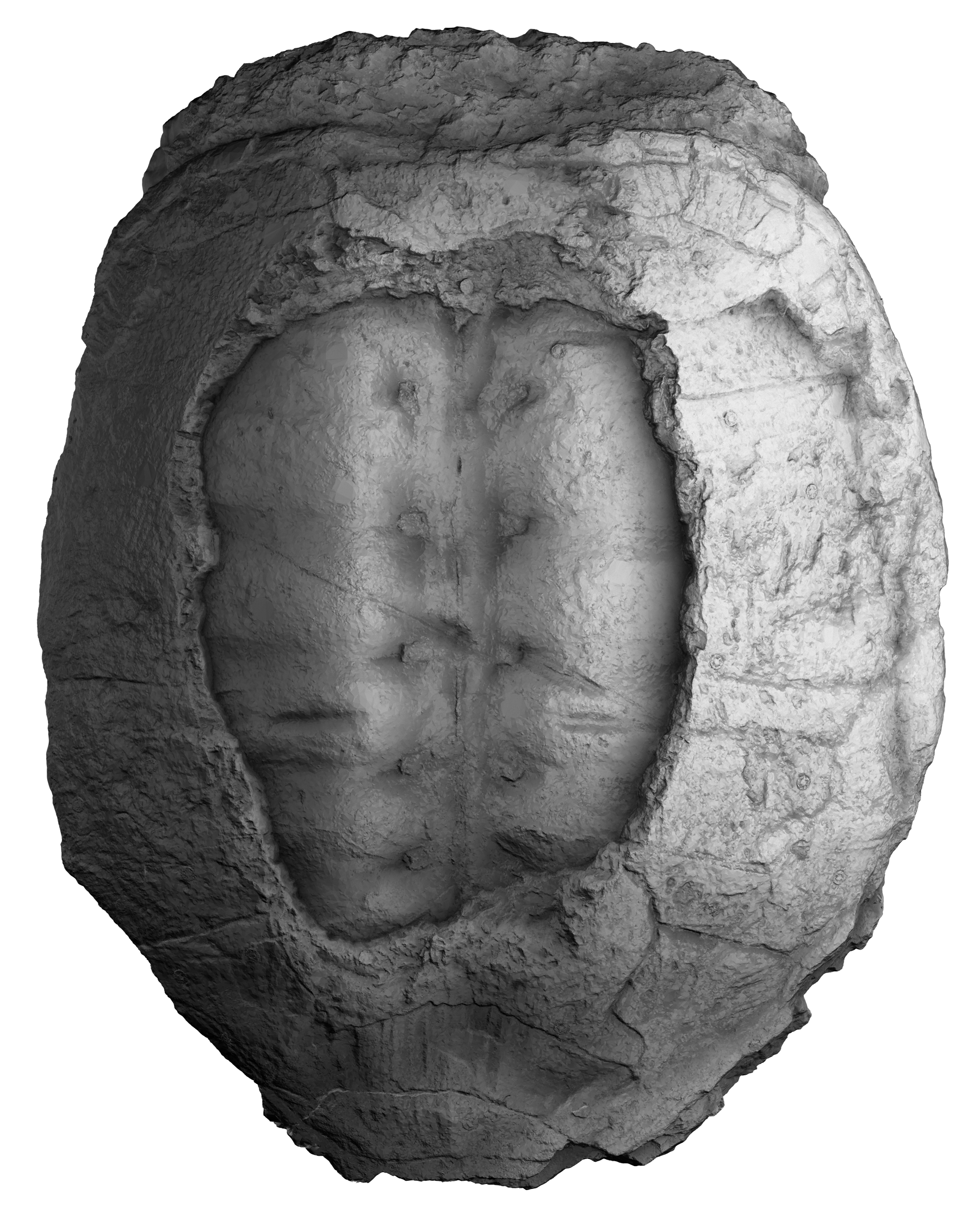

Supplement: Supplemental Information 2 — High resolution 3D surface reconstruction of the carapace of MAJ 2005-11-1, holotype of Plesiochelys etalloni (Pictet & Humbert, 1857). Reconstruction courtesy of and copyright David Vuillermoz, Muse d’archologie du Jura. 3D surface mesh available upon request from the MAJ. [file peerj-02-258-s002.png]

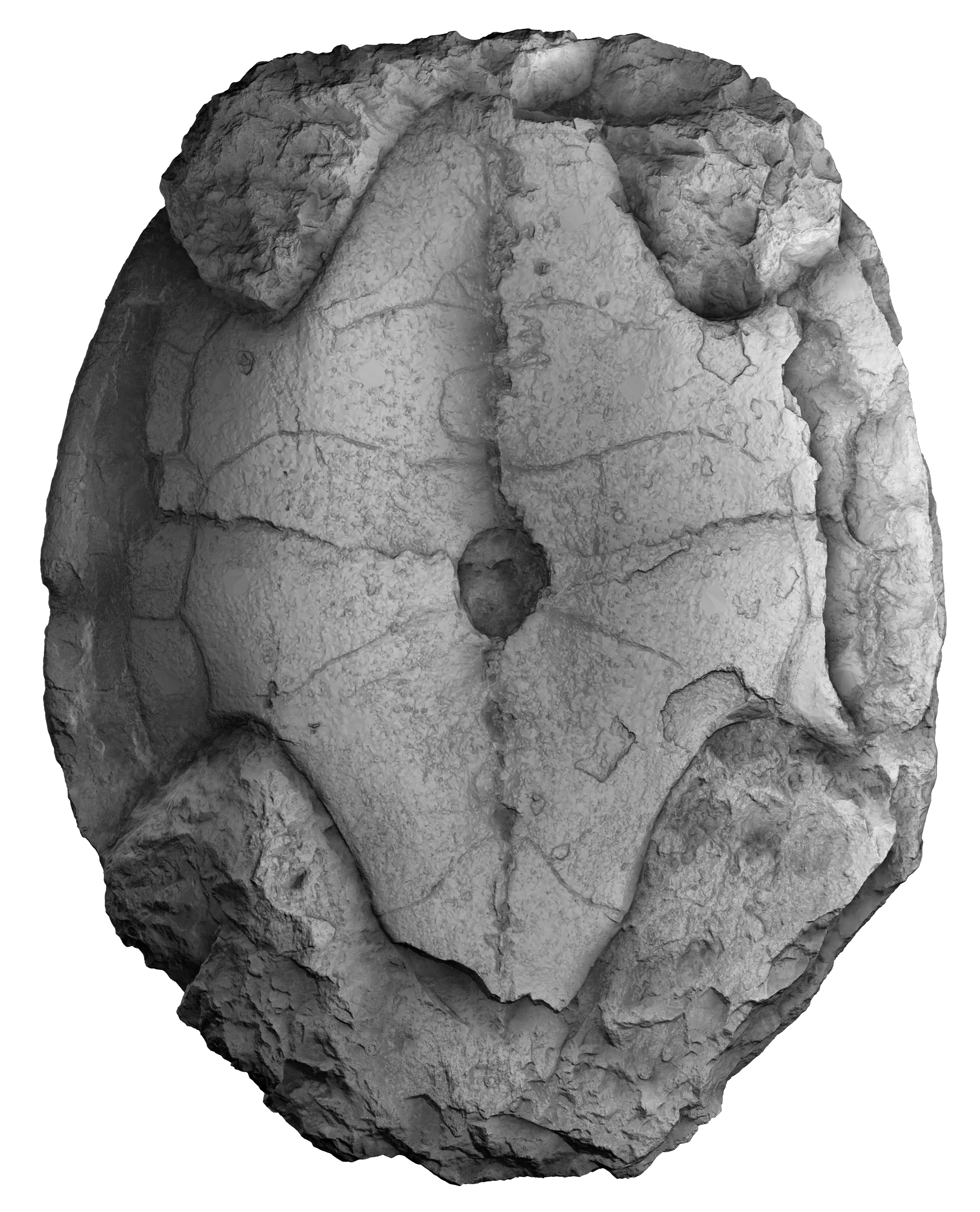

Supplement: Supplemental Information 3 — High resolution 3D surface reconstruction of the plastron of MAJ 2005-11-1, holotype of Plesiochelys etalloni (Pictet & Humbert, 1857). Reconstruction courtesy of and copyright David Vuillermoz, Muse d’archologie du Jura. 3D surface mesh available upon request from the MAJ. [file peerj-02-258-s003.png]
